# Evaluating the performance of Cs_2_PtI_6__−__x_Br_x_ for photovoltaic and photocatalytic applications using first-principles study and SCAPS-1D simulation

**DOI:** 10.1016/j.heliyon.2022.e10808

**Published:** 2022-09-28

**Authors:** Hadeer H. AbdElAziz, Mohamed Taha, Waleed M.A. El Rouby, M.H. Khedr, Laila Saad

**Affiliations:** a Material Science and Nanotechnology Department, Faculty of Postgraduate Studies for Advanced Sciences (PSAS), Beni-Suef University, 62511 Beni-suef, Egypt; b Department of Renewable Energy Science and Engineering, Faculty of Postgraduate Studies for Advanced Sciences (PSAS), Beni-Suef University, 62511 Beni-suef, Egypt

**Keywords:** Perovskite, DFT, Solar cell, Cs_2_PtI_6_, Photocatalysis, SCAPS-1D

## Abstract

All inorganic free-lead halide double perovskites are attractive materials in solar energy harvesting applications. In this study, density functional theory calculations have been used to predict the structures, band structures, and density of states of Cs_2_PtI_6__−__x_Br_x_ with (*x* = 0, 2, 4, and 6). The optical properties (reflectivity, refractive index, absorption, dielectric function, conductivity, and loss function) of these materials have been predicted and discussed. The band edges calculations showed that the Cs_2_PtI_6__−__x_Br_x_ may be an efficient visible-light photocatalyst for water splitting and CO_2_ reduction. The calculated bandgap value of Cs_2_PtI_6_ exhibited a great match with the reported experimental values. It has been seen that increasing the doping content of Br^−^ in Cs_2_PtI_6__−__x_Br_x_ (*x* = 0, 2, 4, and 6) increases the bandgaps from 1.4 eV to 2.6 eV and can be applied in single junction and tandem solar cells. Using Solar Cell Capacitance Simulator (SCAPS), a 1D device modelling has been performed on Cs_2_PtI_6_ inorganic lead-free solar cells. For the fully inorganic device, the effect of replacing organic hole transport materials (HTL) and electron transport materials (ETL) with inorganic ones is investigated while keeping high efficiencies and stabilities of solar cell devices. From the obtained results, it was found that WS_2_ as ETL and Cu_2_O as HTL were the most suitable materials compared to the others. Further investigation studies are performed on the effect of changing metal back contact work function, absorber layer thickness, doping density, and defect density on the power conversion efficiency (PCE) of the solar cell. The optimized suggested structure (FTO/WS_2_/Cs_2_PtI_6_/Cu_2_O/Carbon) obtained a PCE of 17.2% under AM1.5 solar illumination.

## Highlights

• The geometrical structures, electronic, and optical properties of the mixed halide Cs_2_PtI_6_−_x_Br_x_ (x = 0, 2, 4, and 6) were investigated by DFT calculation.
• Cs_2_PtI_6_ material is suitable for single-junction solar cells, while other Br^−^ doping are suitable for top cell in tandem solar cells.
• A device modeling of Cs_2_PtI_6_-based all-inorganic solar cells is provided using SCAPS-1D simulator.
• The results can guide more investigation and optimization of lead-free all-inorganic perovskite solar cells.

## 1 Introduction

The problem of climate change arises from burning fossil fuels which causes the problems of global warming and carbon dioxide (CO_2_) evolutions [1]. Fossil fuel is a limited source of energy as it is depleting from the earth [2, 3]. These crises create a demand for research and development of alternative renewable sources of energy like solar energy harvesting. Solar energy is the cleanest, most abundant, and widely available source of energy. Solar energy is used in photovoltaic (PV) cells to produce electricity and can be consumed by a photocatalysis process to split water into hydrogen (energy vector), oxygen [4, 5], and CO_2_ reduction through photo-chemical reactions [6, 7].

Perovskite materials are emerging in the field of solar energy harvesting because of their unique optical and electronic properties [8, 9, 10]. Many efforts are done for searching for visible light solar-driven water splitting photocatalysis [11, 12] and efficient photovoltaic devices [13, 14]. For photocatalysis applications, good photocatalysis for water splitting or CO_2_ reduction techniques should meet the requirement of (1) a suitable bandgap for absorption of the solar spectrum; (2) immediate electron-hole separation of the material; (3) high stability in aqueous medium against corrosion; (4) the redox potential of the material is suitable for H_2_ generation and CO_2_ reduction. Cs_2_PtI_6_ is also used as a photoanode for the photoelectrochemical (PEC) water oxidation process [15, 16] due to its stability even in an acidic or bases environment. In Jayanthan et al. [15], the heterojunction BiVO_4_/Cs_2_PtI_6_ is used and shows a high quality charge separation from BiVO_4_ to vacancy ordered Cs_2_PtI_6_. The achieved photocurrent density of the BiVO_4_/Cs_2_PtI_6_ junction is 0.92 mA/cm^2^ at a potential of 250 mV. The bare BiVO_4_ gave 0.6 mA/cm^2^ at a potential of 560 mV at 1.23 V (vs.RHE). In Hamdan et al. [16], the Cs_2_PtI_6_ was used as a photoanode for water oxidation in PEC cell. The Cs_2_PtI_6_ photoanode gave a photocurrent density of 0.8 mA/cm^2^ with 12 h of stability. The material also used as an electrocatalyst for hydrogen production at pH∼1 and stable for 6 h 1.23 V (vs.RHE).

For solar cell application, in comparison to conventional silicon solar cells, organic-lead halide perovskite solar cells show high performance. The perovskite solar cell PCE begin from 3.8% in 2009 [17] up to 25.5% in 2021 for single junction and 29.15% for perovskite/Si tandem two terminal cells [18]. Organic compounds are degradable which affects the stability of the solar cell. Another problem that limits the spreading of perovskite solar cells is the lead (Pb) element toxicity. Many efforts have been done to replace conventional organic (CH_3_NH_3_^+^, CH(NH_2_)_2_^+^) with inorganic (K^+^, Rb^+^, Cs^+^) [19, 20, 21, 22, 23, 24]. One of the high-efficiency candidates of inorganic solar cells is CsPbI_3_ [25, 26] which has an efficiency of around 20% [27] due to the slightly wide bandgap of CsPbI_3_ ∼1.7 eV and the presence of lead. CsSnI_3_ has a band gap of ∼1.3 eV which is ideal for capturing solar illumination [28] but suffers from low stability and the Sn^+2^ element oxidizes to Sn^+4^ forming the air-stable compound Cs_2_SnI_6_ [29]. This phenomenon attracts researchers to study the A_2_BX_6_ family perovskite to tackle the problem of instability and give alternatives for lead element. The drawback of Cs_2_SnI_6_ in solar cell fabrication is the lack of efficiency [30]. The maximum efficiency of Cs_2_SnI_6_ based solar cell is around 1.5% and 2.025% for mixed halide Cs_2_SnI_4_Br_2_ based solar cell [31]. One of the promising materials of A_2_BX_6_ family is Cs_2_PtI_6_. Cs_2_PtI_6_ is considered as a fully inorganic lead free double halide perovskite material. Cs_2_PtI_6_ crystallizes in the form of Fm$\bar{3}$m K_2_PtCl_6_ cubic structure form [32]. The stability of Cs_2_PtI_6_ material is up to 60 days in ambient conditions and 40 days in aqueous solution [33] and at high temperature [34]. It has a relatively high lifetime carrier of 2 μs and a low defect density of 2.5×10^12^ cm^−3^. Two prototypes of full device perovskite solar cell were fabricated with efficiencies of 10% and 13.88% respectively using ethylene diamine treatment [34]. Bromine (Br^−^) doping and substitution applied to many perovskites-based solar cells for tuning bandgap and increasing stability [35, 36, 37]. The Br^−^ doping affects the band gap of the structure and this in return allows the materials to be used in different photocatalysis water splitting and CO_2_ reduction applications.

One dimensional solar cell capacitance simulation program SCAPS-1D developed by Burgelman et al. in gent university [38, 39] considered as one-dimensional simulation tool that solves the continuity and Poisson equations for semiconductors. SCAPS-1D can model up to seven layers of solar cell device with the ability to modify the properties of every layer separately. The calculation results output can include current-voltage, capacitance-voltage, capacitance frequency, and quantum efficiency characteristics diagrams. The proper choice of ETL and HTL materials has a good impact on solar cell performance and stability. The most used HTL material for perovskite solar cell is 2,2′,7,7′-tetrakis-(N, N-di-4-methoxyphenylamino)-9,9′-spirobifluorene (spiro-OMeTAD). Spiro-OMeTAD material suffers from lack of stability due to degradable organic elements besides it is expensive [40]. Other cuprous based HTL materials like cuprous oxide [41] and cuprous iodide [42] are integrated with PSC with high performance. Other HTL materials are investigated like MoO_3_ [43] for acquiring high performance, high stability, and low cost. ETL materials are important for the efficient electron collecting process to increase the PCE of solar cell. ETL material should maintain high electron mobility, chemical, and photostability. Another important feature is to be fabricated easily with perovskite materials. Indium gallium zinc oxide (IGZO) [44], zinc selenide (ZnSe) [45], and tungsten disulfide WS_2_ [46] attracted many researchers for their high conductivity and high mobility as an n-type semiconductor.

In this work, the structural, electronic, and optical properties of Br^−^ doped Cs_2_PtI_6_ were performed by density functional theory (DFT) calculations. The suitability of these materials, as its possibility to be used as photocatalyst for water splitting and CO_2_ reduction processes was evaluated for the first time based on our best knowledge. The Cs_2_PtI_6_ material was simulated as the main absorber layer of a solar cell device. An investigation of different suitable inorganic HTL and ETL materials was done. The HTL materials are Cu_2_O, CuI, MoO_3,_ and spiro-OMeTAD organic HTL to compare with inorganic ones. The ETL materials are (IGZO, WS_2_, ZnSe, and CdS). The effect of the back contact work function on the performance of PSC is investigated. Furthermore, the change of thickness, doping, and defect density of Cs_2_PtI_6_ material are optimized for maximum PCE.

## 2 DFT study

### 2.1 Computational details

The structure of Cs_2_PtI_6_ has a face-centered cubic structure with a space group of Fm$\bar{3}$m (No. 225) [47]. The studied structures with Br^−^ doping were optimized using the Cambridge Serial Total Energy Package (CASTEP) program, which implements DFT [48]. The bandgap and the lattice parameter constants of Cs_2_PtI_6_ were calculated with local density approximation (LDA) [49] Perdew and Alex Zunger (CA) [50]-Perdew and Alex Zunger (PZ) [51] functional and the generalized gradient approximation (GGA) functionals (Perdew Burke-Ernzerhof (PBE) [52], revised PBE (RPBE) [53], revised PBE functional for solids (PBESOL) [54], Wu-Cohen (WC) [55], and Perdew-Wang 91 (PW91) [56]), as well as the hybrid Heyd-Scuseria-Ernzerh (HSE06) functional. Norm-conserving pseudopotential was used for the calculation of the interaction between the ionic core and valence electrons. The k-Monkhorst-Pack was set to 3 × 3×3 along the Brillouin zone of the material structure. The cut-off energy is set to 480 eV for HSE06 calculations and 400 eV for the LDA/GGA functionals. The Broyden Fletcher–Goldfarb Shannon (BFGS) algorithm is used with convergence tolerance energy of 2 × 10^−5^eV per atom. The convergence criteria of the interaction forces between atoms were set to 0.05 eV/Å, and the convergence criteria of maximum displacement of atoms are set to 0.002 Å. The optical properties of the Cs_2_PtI_6__−__*x*_Br_*x*_ were calculated by the RPBE method by energy calculations on the optimized unit cells obtained from the HSE06 calculations. The band gaps of the Cs_2_PtI_6__−__*x*_Br_*x*_ obtained from the HSE06 method were used in the optical (reflectivity, absorption, refractive index, dielectric function, conductivity, loss function) calculations using the scissors operator, as implemented in the CASTEP code.

The theoretical predictions of the bottom of the conduction band (E_CB_) and the top of the valence band (E_VB_) edges of the Cs_2_PtI_6__−__*x*_Br_*x*_ (*x* = 0, 2, 4, and 6) were calculated according to Butler and Ginley [57]. The method mainly depends on Mullikens equations, which calculate the values of electron negativity, electron affinity, E_VB_ and E_CB_. Mullikens defined the electron affinity of an atom, Eq. (1), as the arithmetic mean of atomic electron affinity A_f_ tabulated in [58] and the first ionization energy I_1_ tabulated in [59].

(1) $$\chi_{atom}^{M}=\;\frac{1}{2}\left(A_{f}+\;I_{1}\right)\;$$

For a compound with 3 elements p, q and r with number of atoms l, m and respectively for each, [60, 61]. The electron negativity for a compound can be calculated using Eq. (2) as: -

(2) $$\chi_{compound\;}^{M}=\;{\left(\chi_{p}^{l}\;.\;\chi_{q}^{m}\;.\;\chi_{r}^{n}\;\right)}^{\frac{1}{l+m+n}}$$

Applying this equation to Cs_2_PtI_6_ material we obtain Eq. (3):

(3) $$\chi_{Cs_{2\;}Pt\;I_{6\;}}^{M}={\left(\chi_{Cs}^{2}\;.\chi_{Pt\;}^{1}.\chi_{I}^{6}\right)}^{\frac{1}{9}}$$

Electron affinity χ of a compound can be calculated via Eq. (4) as:-

(4) $$\chi =\;\chi M-\;\frac{1}{2}\;E_{g}\;$$

the theoretical predictions of E_CB_ and E_VB_ of the Cs_2_PtI_6__−__*x*_Br_*x*_ (*x* = 0, 2, 4, and 6) were calculated using Eqs. (5) and (6) [62, 63], respectively.

(5) $$E_{CB\;}=\;\chi atom-\;E^{e}-0.5\;E_{g}$$

where *E*^e^ is the energy of the free-electron on the hydrogen scale (4.44 ± 0.02 eV), *E*_g_ is the bandgap energy calculated from the HSE06 method. χ is the Mulliken's electronegativity of the semiconductor. The Mulliken’s electronegativity (χ^atom^) of a neutral atom, is defined as the arithmetic mean of the atomic electron affinity and the first ionization energy:

(6) $$E_{VB\;}=\;E_{CB}+\;E_{g}$$

### 2.2 Structure properties

**Figure 1:**
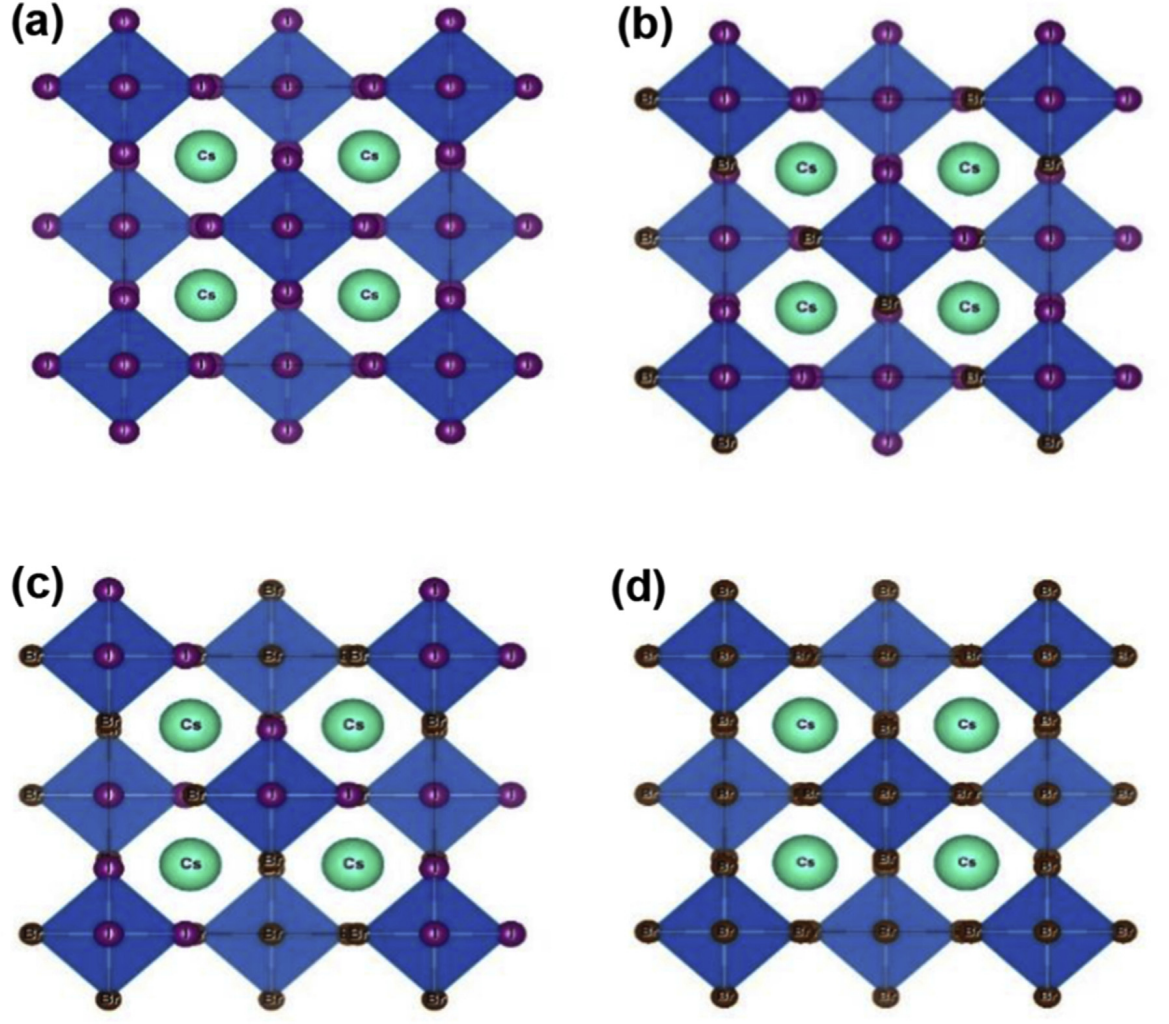
The crystal structures of (a) Cs_2_PtI_6_, (b) Cs_2_PtI_4_Br_2_, (c) Cs_2_PtI_2_Br_4_, and (d) Cs_2_PtBr_6_.

The optimized crystal structures of Cs_2_PtI_6__−__*x*_Br_*x*_ perovskites are shown in Figure 1. The pure structure is shown in Figure 1(a) Cs_2_PtI_6_ *x* = 0 and Figure 1(d) Cs_2_PtBr_6_ (*x* = 6), respectively. The I to Br ratio in the structure Cs_2_PtI_6__−__*x*_Br_*x*_ is represented by *x* = 2 for Cs_2_PtI_4_Br_2_ Figure 1(b) and *x* = 4 for Cs_2_PtI_2_Br_4_ Figure 1(c). As shown in Table 1, the bandgap of Cs_2_PtI_6_ is calculated using different DFT functionals. The GGA functionals used were PBE, RPBE, WC, PBESOL, and PW91 with resultant bandgap values of 1, 1.4, 0.8, 0.8, 1, and 0.5 eV, respectively, and LDA (CA-PZ) with a bandgap of 0.5 eV. These band gap values are not agreed with the experimental band gap (1.4 eV [34]), except for the RPBE functional. It is well known that the GGA/LDA functionals overestimate the band gap value, and probably the obtained band gap by the RPEB functional is just an artifact since it provides the worst lattice parameters among all tested functionals. The hybrid HSE06 has successfully predicted the experimental band gap (1.4 eV).

**Table 1:** The calculated lattice constant and bandgap of Cs_2_PtI_6_.

Functional	GGA					LDA	HSE06	Experimental
	PBE	RPBE	WC	PBESOL	PW91	(CA-PZ)		
Bandgap (eV)	1	1.4	0.8	0.8	1	0.5	1.4	1.4 [34] 1.37 [33]
Lattice A°	8.3	8.9	8.06	8.05	8.2	7.8	7.9	8.03 [47]

The reported experimental value of the lattice constant of Cs_2_PtI_6_ is 8.03 Å [32] which agrees with the HSE06 calculated lattice constant value as shown in Table 2 with an acceptable error of ∼1.6 %. The optimized lattice constant of Cs_2_PtBr_6_ is 7.3 Å with an error of 3.2 % from the experimental measured value [64]. It is noticed that the Br^−^ doping content increase reduces the lattice constant and increases the bandgap gradually.

**Table 2:** The calculated lattice parameter and bandgap of Cs_2_PtI_6__−__x_Br_x_ using HSE06 functional versus experimental values.

Material	HSE06		Experimental	
Parameter	Lattice	*E*_g_ (eV)	lattice	*E*_g_ (eV)
Cs_2_PtI_6_	7.9	1.4	8.03 [32]	1.4 [34] 1.37 [33]
Cs_2_PtI_4_Br_2_	7.7	1.6	-	-
Cs_2_PtI_2_Br_4_	7.4	1.7	-	-
Cs_2_PtBr_6_	7.3	2.6	7.54 [64]	-

### 2.3 Electronic properties

**Figure 2:**
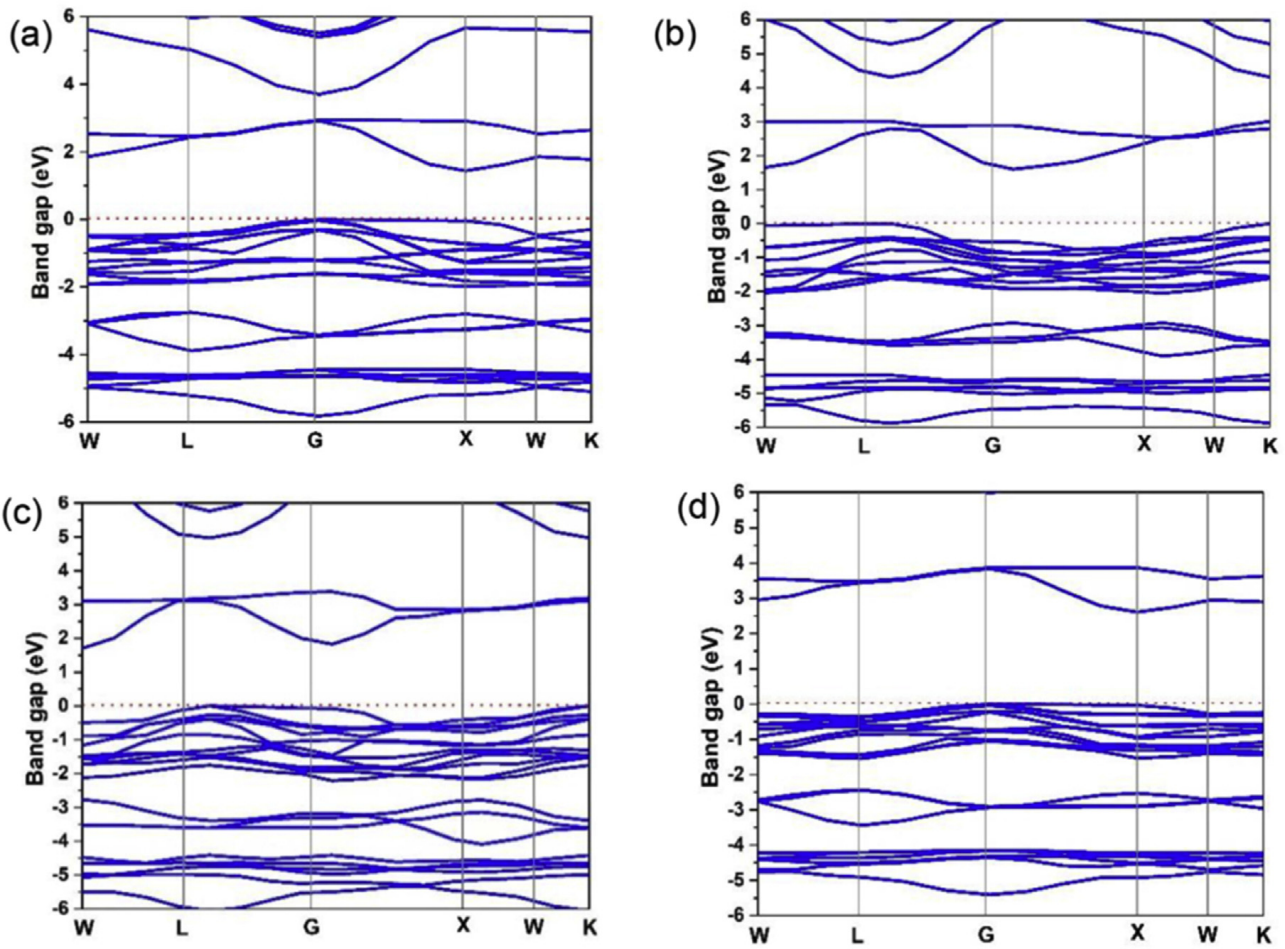
The band structures of (a) Cs_2_PtI_6_, (b) Cs_2_PtI_4_Br_2_, (c) Cs_2_PtI_2_Br_4_, and (d) Cs_2_PtBr_6_ by using the HSE06 functional.

The calculated band structures of Cs_2_PtI_6__−__x_Br_x_ are shown in Figure 2 using HSE06 functional. The calculated values of band gap of Cs_2_PtI_6__−__x_Br_x_ are listed in Table 1, The calculated bandgap of Cs_2_PtI_6_ with HSE06 functional is 1.4 eV which agrees well with the experimental bandgap values of (1.4 eV [34] and 1.37 eV [33]). Till date, no experimental data are provided for the bandgap values of Cs_2_PtI_4_Br_2_, Cs_2_PtI_2_Br_4_, and Cs_2_PtBr_6_. The Cs_2_PtI_4_Br_2_ has a bandgap of 1.6 eV, the Cs_2_PtI_2_Br_4_ has a bandgap of 1.7 eV and Cs_2_PtBr_6_ has a bandgap of 2.6 eV.

**Figure 3:**
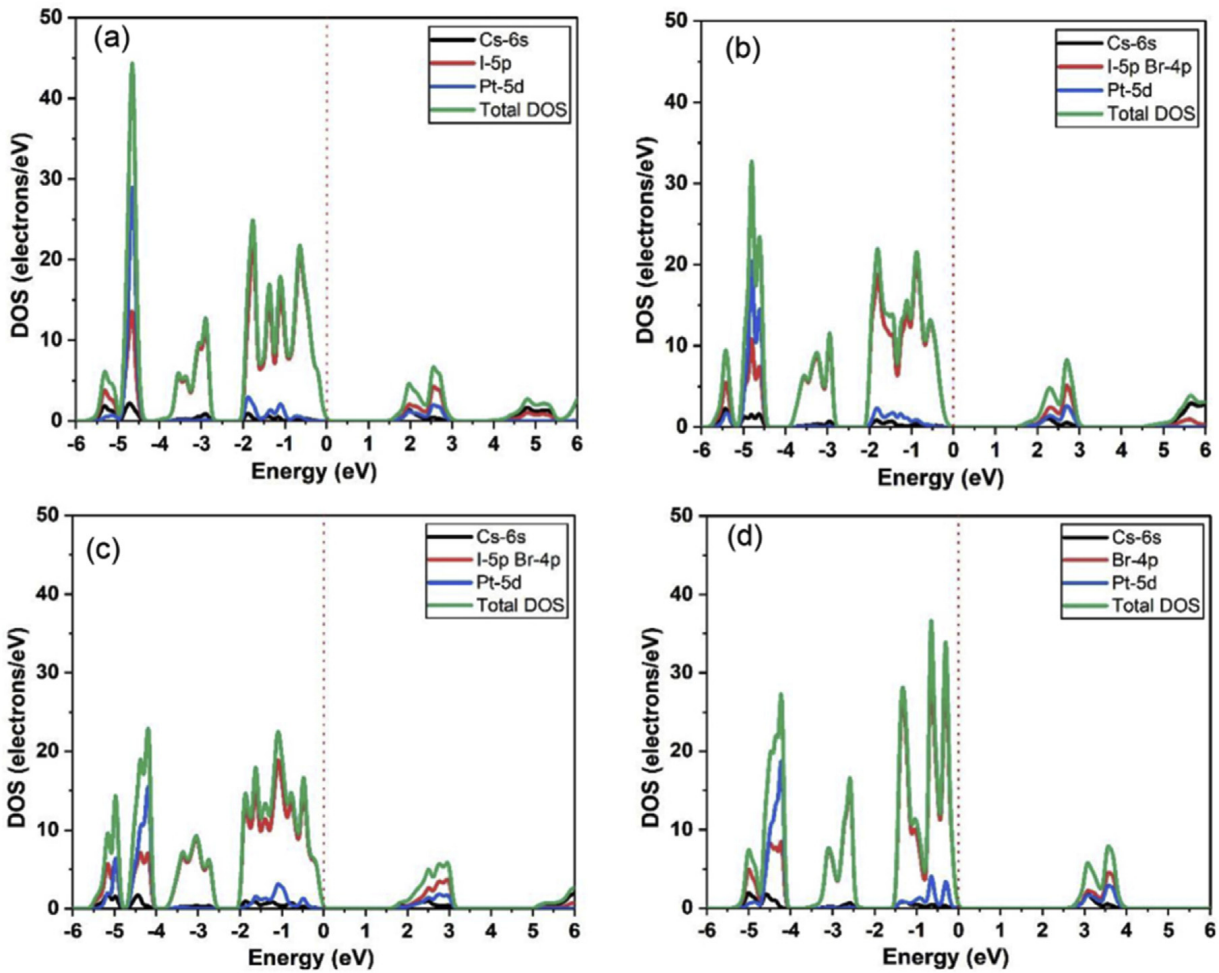
Total and partial densities of states for (a) Cs_2_PtI_6_, (b) Cs_2_PtI_4_Br_2_, (c) Cs_2_PtI_2_Br_4_, and (d) Cs_2_PtBr_6_ using the HSE06 functional.

Figure 3 shows the total density of states (DOS) and partial density of states (PDOS) of Cs_2_PtI_6__−__x_Br_x_ using HSE06 functional. Fermi level position is dedicated by the dotted vertical red line at zero energy level. The PDOS of pure halide materials (Cs_2_PtI_6_ and Cs_2_PtBr_6_) show the dominance of I-5p orbitals for the valence band maximum (VBM) and slight dominance of Cs – 6s and Pt-5d on the conduction band minimum (CBM), as shown in Figure 3(a,d). The PDOS for mixed halide materials (Cs_2_PtI_4_Br_2_ and Cs_2_PtI_2_Br_4_) show that the dominance in the VBM shared by or I-5p and Br -4p orbitals, while Cs-6s and Pt-5d have slight dominance in the CBM, Figure 3(b,c).

### 2.4 Optical properties

**Figure 4:**
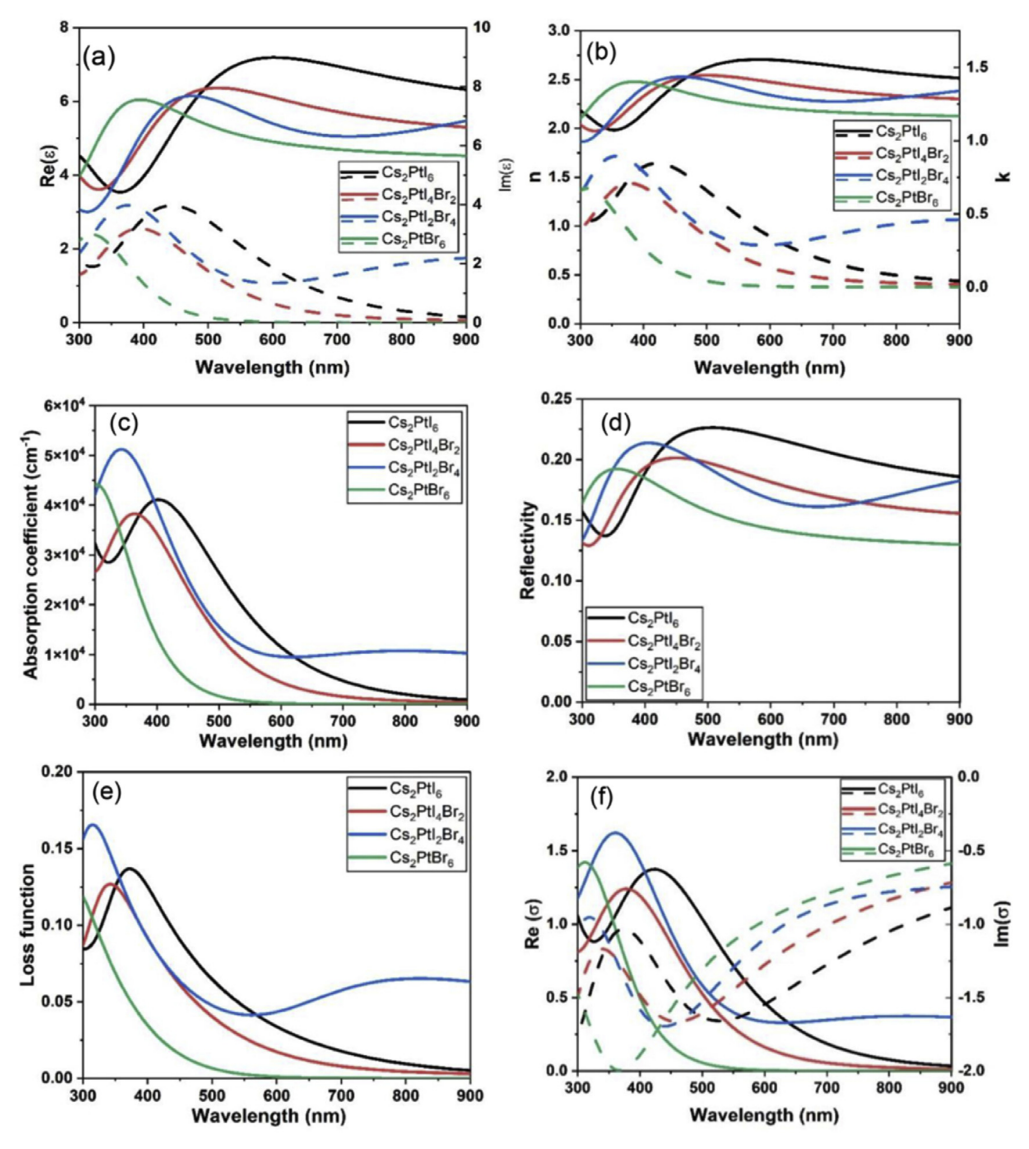
The optical properties of Cs_2_PtI_6__−__x_Br_x_ (a) real (solid lines) and imaginary part (dashed lines) of dielectric function, (b) real (solid lines) and imaginary part dashed lines of refractive index, (c) Absorption coefficient, (d) Reflectivity, (e) Loss function and (f) real (solid lines) and imaginary part (dashed lines) of conductivity.

The dielectric function is expressed as $\varepsilon_{\mathrm{material}}=\;\varepsilon^{\prime }-i\;\varepsilon^{\prime \prime }$ where $\varepsilon^{\prime }$ is the real part of dielectric function and $\varepsilon^{\prime \prime }$ is the imaginary part. Figure 4(a) shows the real and imaginary part of the dielectric function versus the wavelength of the solar spectrum. The real part of the dielectric function of Cs_2_PtI_6__−__x_Br_x_ is relatively high in the visible spectrum. The peaks of Cs_2_PtI_6_ are at λ = 300 nm and λ = 550 nm respectively. Increasing the Br^−^ content causes a blue shift on the dielectric function spectrum. The peak of the real part of the dielectric function of Cs_2_PtBr_6_ is at λ = 350 nm. The imaginary part of the dielectric function shows a blue shift increase with increasing Br^−^ content. This means that the absorption of these materials can be tuned by Br^−^ content doping. Figure 4(b) shows the real *n* and imaginary *k* part of the refractive index as a function of wavelength. The real refractive index of Cs_2_PtI_6_ increases gradually and then saturates for >500 nm with values of 2.5. Increasing Br^−^ content lowers the *n* values with a blue shift. Higher values of *n* indicate a good absorption of the material to the incident spectrum. Extinction coefficient (k) indicates how the incident radiation attenuates through the material. Like the imaginary part of the dielectric function, increasing Br^−^ content shows a blue shift in the *k* values versus wavelength. The higher values of *k* of these materials are for λ between 350 and 450 nm in the middle of the spectrum which means high absorption of radiation by the materials in this region. The absorption coefficient versus wavelength is shown in Figure 4(c). The peak of the absorption coefficient of Cs_2_PtI_6_ is at λ = 400 nm. The Br^−^ content has a blue shift on the curve peak so it shifted from 400 nm for Cs_2_PtI_6_ to 300 nm for Cs_2_PtBr_6_. Among these materials, Cs_2_PtI_2_Br_4_ shows a strong peak of absorption coefficient at λ = 350 nm Figure 4(d) shows the reflectivity versus wavelength for the Cs_2_PtI_6__−__x_Br_x_ perovskites. The reflectivity is used to study the surface properties of a material, which is equal to the ratio of the reflection to the incident power. These materials show low reflectivity all over the spectrum. The loss functions for Cs_2_PtI_6__−__x_Br_x_ were calculated and shown in Figure 4(e). The results obtained show that the loss function of the four materials is smaller than 1 which means low values for the loss function all over the solar spectrum. Figure 4(f) shows the frequency dependent optical conductivity σ (ω) = σ_1_ + i σ_*2*_(ω)*.* The complex σ (ω) is derived from the complex dielectric function as described in Eqs. (7) and (8) [65].

(7) $$\sigma_{1\;}=\;\omega \;\varepsilon^{\prime \prime }\varepsilon_{0}$$

(8) $$\sigma_{2\;}=\;\omega \;\varepsilon^{\prime }\varepsilon_{0}$$

where $\sigma_{1\;}\;$ and $\sigma_{2\;}\;$ are real and imaginary part of optical conductivity, $\varepsilon_{0}\;$ is the dielectric constant of free space and ω is the angular frequency. The real part of optical conductivity spectrum shows a blue shift increase with Br^−^ doping. The imaginary part of the optical conductivity shows a red shift increase with Br^−^ doping.

### 2.5 Evaluation of the Cs_2_PtI_6__−__x_Br_x_ materials for photocatalysis and photovoltaic applications

#### 2.5.1 Photocatalytic H_2_, O_2_ evolution, and CO_2_ reduction process

**Figure 5:**
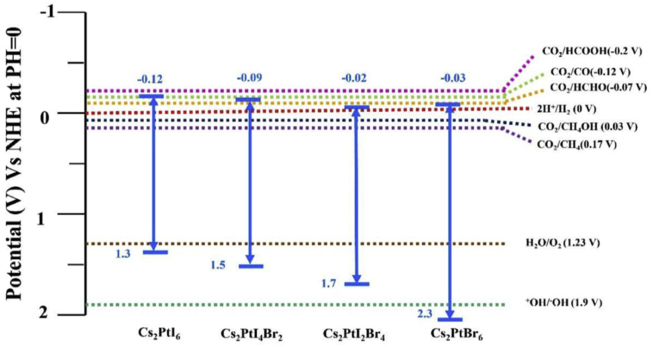
Band edges of Cs_2_PtI_6__−__x_Br_x_ (x = 0, 2, 4, 6) compared to the redox potentials of the water-splitting reaction and CO_2_ reduction photodegradation processes at normal hydrogen electrode (NHE) scale at pH = 0.

Under solar illumination, the incident photons with energies higher than or equal to the bandgap of the semiconductor, the electrons in the valence band of the semiconductor excited to the conduction band leaving a positive hole. The photo-generated electron-hole pairs will move to the surface of the semiconductor and cause redox reactions. Figure S1 (Supplementary Information) shows the photocatalytic process under solar illumination. The determination of bandgap edges of the semiconductor is a key factor in the photocatalytic operation process. The bandgap edges positions define the thermodynamic limitations of the photochemical reaction caused by the photo-generated electron-hole pair of the semiconductor. The efficient photocatalytic water splitting semiconductor material should meet the criteria of (1) The semiconductor’s conduction band minimum should be more negative than the redox potential of H^+^/H_2_ (0 V vs NHE) and (2) the valence band maximum should be more positive than the potential edge of O_2_/H_2_O. These criteria apply to the CO_2_ reduction photocatalytic process. The E_CB_ and E_VB_ values of Cs_2_PtI_6_, Cs_2_PtI_4_Br_2_, Cs_2_PtI_2_Br_4_ and Cs_2_PtBr_6_ are shown in Figure 5. It is noticed that the E_CB_ values are smaller than 0 V (the redox potential of H_2_) for the materials and the E_VB_ values are greater than 1.23 V (the potential of O_2_). Thus, the Cs_2_PtI_6__−__x_Br_x_ materials will be suitable for photo-catalytic water splitting. For CO_2_ reduction process, it is shown that all the materials E_VB_ are more negative than the CO_2_/CH_4_OH and CO_2_/CH_4_. This means that the materials may be suitable for CO_2_ reduction to produce CH_4_OH and CH_4_. The E_VB_ level of Cs_2_PtI_6_ is at −0.12 V which means that the material may be suitable for CO_2_ reduction to HCHO. Cs_2_PtI_4_Br_2_ material also may reduce CO_2_ to HCHO. The estimation of electron negativity, electron affinity and band positions are shown in supplementary information Table S1.

#### 2.5.2 Photovoltaic device

From the DFT study, it is concluded that, All the four materials are direct bandgap semiconductors. Direct bandgap semiconductors are more efficient to be applied for solar cell devices than indirect band gap semiconductors. According to Shockley–Queisser limit [66], to maintain high power conversion efficiency of a single junction solar cell, the best band gap efficiency of a material should be in the range of 1.3–1.4 eV [67]. The Cs_2_PtI_6_ has a bandgap of 1.4 eV which is suitable for single junction perovskite solar cell.

## 3 Numerical analysis of all-inorganic perovskite Cs_2_PtI_6_ solar cell device using SCAPS-1D

To the best of our knowledge, there is no report on the numerical analysis of Cs_2_PtI_6_-based all-inorganic perovskite solar cells PSCs. Therefore, further studies are required to figure out the optimum combination of different materials to be used as various layers in the Cs_2_PtI_6_-based PSC. In this work, a detailed investigation of Cs_2_PtI_6_-based PSCs is conducted. First, the validity of the simulation was verified by comparing experimental results with the simulation results. Since there is much room for further performance enhancement, the effects of different parameters (ETL and HTLlayers, back metal contact, acceptor carrier and defect density concentrations) on the cell performance are investigated. In addition to determining the optimum thickness of the Cs_2_PtI_6_ active layer. Finally, the optimum PSCs cell structure was elected using the appropriate layers regarding the results of this study.

### 3.1 The basic simulation parameters

Table S2 (sup. information) summarizes the physical parameters of the simulated Cs_2_PtI_6_ solar cell device. The working temperature of the device is assumed to be as the actual experimental conditions 298.15 K equivalent to regular room temperature. The series resistant Rs is set to be 14 (Ω.cm^2^) and the illumination is based on reference air mass AM1.5 spectra. The range of wavelength of the spectra is set to be in the range of 300–900 nm. The Cs_2_PtI_6_ based solar cell device is modelled as separate different seven layers. The front contact is modelled as a flat band with surface recombination velocity of electrons and holes are 10^5^ cm/s and 10^7^ cm/s, respectively. The metal back contact is modelled using different materials with different work functions and surface recombination velocity of electrons and holes are set to 10^7^ cm/s and 10^5^ cm/s respectively. The simulated device structure consisted of fluorine-doped tin oxide (FTO) as front contact followed by an ETL, perovskite, HTL layers and a metal back contact, as shown in Figure S2 (a). The solar cell device layers are modelled with two embedded interface defect layers (IDL); one between ETL and Cs_2_PtI_6_ active layer and the other is the back IDL between Cs_2_PtI_6_ and HTL layer. The front and back IDLs are ultra-thin 5 nm layers with the same physical parameters of the absorber active layer and used to determine the effect of the interface recombination. The physical parameters of FTO, IDLs and Cs_2_PtI_6_ are listed in Table S3. Physical parameters of different HTL and ETL materials are shown in Tables S4 and S5, respectively [34, 43, 47, 68, 69, 70, 71, 72, 73, 74, 75]. Metal contacts work function are shown in Table S6 [76, 77, 78, 79, 80, 81]. The defect density N_t_ can be calculated from the bulk lifetime model, Eq. (9).

(9) $$N_{t}=\;\frac{1}{\sigma \;\tau \;V_{th\;}}$$

where the V_th_ thermal velocity of electrons and holes is 107 cm/s, The capture cross section of electrons and holes σ are estimated to be 2 × 10−14 cm2. The carrier lifetime τ value of Cs_2_PtI_6_ is >2 μs. The initial estimated value of Nt is found to be 2.5×1012 cm^−3^, and the energetic distribution is assumed to be single.

### 3.2 Model device verification

Firstly, the model was verified with previously published experimental data reported by Schwartz *et al.* [34]. The typical reference-cell structure is FTO/CdS/Cs_2_PtI_6_/Spiro-OMETAD/Carbon. The CdS and Spiro-OMETAD are the initial ETL and HTL materials, respectively. The resulted current density-voltage (J-V) characteristics diagram of the solar cell with structure FTO/CdS/Cs_2_PtI_6_/Spiro-OMETAD/Carbon show a short circuit current density (Jsc) of 20.1 mA/cm^2^, open circuit voltage (V_oc_) of 1.12 V, power conversion efficiency (PCE) of 10.16 % and fill factor (FF) of 44%, as shown in Figure S2 (b). These results are in a good agreement with the experimental results (the values are Jsc = 20 mA/cm^2^, Voc = 1.12 V, PCE = 10.06 % and FF = 41%) [34]. The difference between numerical and experimental values is in the acceptable range.

### 3.3 Bandgap alignment for ETL and HTL

**Figure 6:**
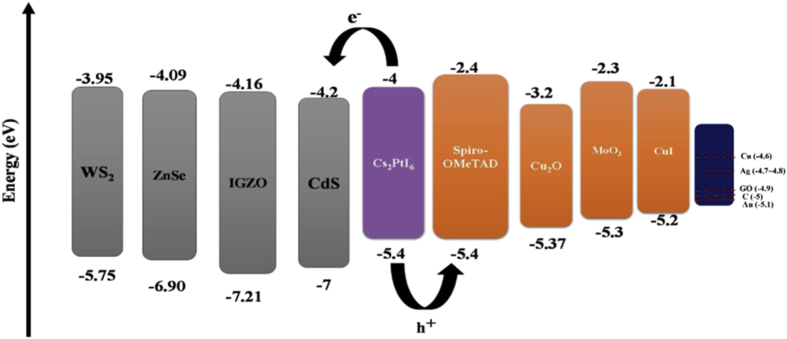
Band gap alignment of Cs_2_PtI_6_ material with different ETLs and HTLs.

The ETL and HTL materials should be chosen with suitable band edges to match the CBM and VBM of the active layers to obtain a high-efficiency solar cell. The proposed bandgap alignment schematic diagram for different ETL and HTL materials with respect to Cs_2_PtI_6_ active layer is shown in Figure 6. All the HTL materials' highest occupied molecular orbital (HOMO) levels aligned well with the valence band level of Cs_2_PtI_6_ material and the ETL lowest unoccupied molecular orbital (LUMO) level aligned well with the conduction band of the Cs_2_PtI_6_ material. The CBM, VBM of used ETL and HTL materials are stated from previously published experimental and theoretical work research. In order to maintain high efficiency PSC the choice of ETL material should meet the criteria.

(i) The mobility of electrons should be high to maintain fast electron transport within the ETL.
(ii) CBM alignment with the active material Cs_2_PtI_6_

For HTL materials it should meet the criteria.

(i) High chemical stability to environmental conditions
(ii) The hole mobility is good enough for fast transfer of the holes to the electrode.
(iii) Blocking the transfer of electrons to the anode.
(iv) The highest occupies molecular orbital (HOMO) should be higher than the VBM of perovskite layer to ensure the process of photogenerated holes extraction.

### 3.4 Effect of HTL materials

**Figure 7:**
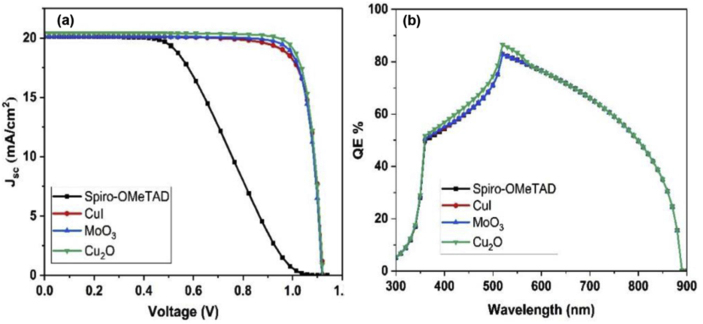
(a) J-V measurement and (b) QE curves for Cs_2_PtI_6_-based solar cells assembled with different HTL materials.

The ETL layer was fixed to be CdS however, Spiro-OMeTAD was used as a reference HTL materials, in addition to other three different materials namely MoO_3_, Cu_2_O and CuI were simulated to study the effect of HTL layer properties on the solar cell performance. J-V characteristics diagram and QE measurements with different HTLs are shown in Figure 7(a,b), respectively. The Cu_2_O HTL has high hole mobility which in turns gives high performance, the solar cell device using Cu_2_O material has PCE values of 14.2%, Voc of 1.12 V, Jsc of 20.4 and FF of 62% as shown in Table 3. The QE values are high all over the spectrum as the assumption of zero reflection at each layer of the device for calculations simplicity. The QE spectrum shows negligible change with different HTL materials. The reason of this is because the HTL materials are placed in the back of the structure and their optical absorptions are not significant.

**Table 3:** Solar cell parameters with different HTL materials.

Structure	J_sc_ (mA/cm^2^)	V_oc_ (volts)	FF %	PCE %
FTO/CdS/Cs_2_PtI_6_/Spiro-OMETAD/C	20.1	1.12	44	10.16
FTO/CdS/Cs_2_PtI_6_/MoO_3_/C	20.14	1.11	61	13.9
FTO/CdS/Cs_2_PtI_6_/Cu_2_O/C	20.4	1.1	62	14.2
FTO/CdS/Cs_2_PtI_6_/CuI/C	20.13	1.12	60	13.7

### 3.5 Effect of ETL materials on the cell performance

**Figure 8:**
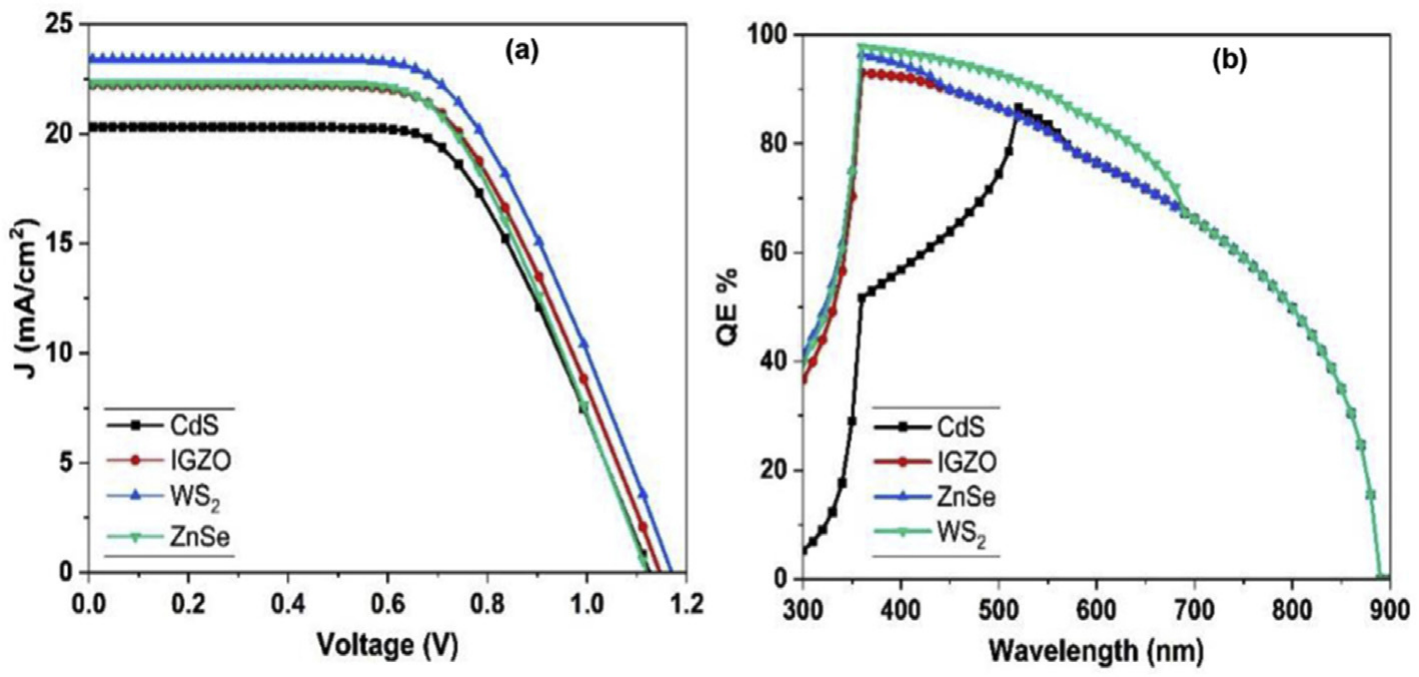
(a) JV measurement and (b) QE curves for Cs_2_PtI_6_-based solar cells assembled with different ETL materials.

In this section the HTL layer was fixed to be Cu_2_O, since it shows the best appropriate performance with Cs_2_PtI_6_ active layer, as discussed in the previous section (section 3.4). Regarding to the ETL material, three different promising materials are selected and simulated namely, indium gallium zinc oxide (IGZO), tungsten sulphide (WS_2_) and zinc selenide (ZnSe). These materials are expected to show good performance as they have a good matching with Cs_2_PtI_6_ active layer as illustrated in the band alignment schematic diagram, Figure 6. Changing ETL material has a significant effect on the value of both Jsc and Voc. For this type of cells CdS ETL layer has found to have the lowest values of Jsc and Voc compared to other selected ETL materials. However, IGZO and ZnSe ETL layers have similar effect in the Cell performance as described in Figure 8(a). WS_2_ ETL layer has a remarkable change over other ETL materials, and this may be due to Ws_2_ has high electron mobility value [46], the ability of absorbing wide range of solar light (Eg∼1.8 eV) and the good band alignment with Cs_2_PtI_6_. The QE values are relatively high for all the simulated ETL materials, as we considered zero reflection of the solar cell layers for simplicity. The QE values are the highest using WS_2_ as ETL compared to other materials Figure 8(b). For Cs_2_PtI_6_-based cell using WS_2_ as ETL layer has the best effect on the cell efficiency, Jsc of 23.4 mA/cm^2^, Voc of 1.13 V, FF of 58.5% and PCE values of 15.4%, as shown in Table 4.

**Table 4:** Solar cell parameters with different ETL layers.

Structure	J_sc_ (mA/cm^2^)	V_oc_ (volts)	FF %	PCE %
FTO/IGZO/Cs_2_PtI_6_/Cu_2_O/C	22.2	1.13	59.2	14.8
FTO/ZnSe/Cs_2_PtI_6_/Cu_2_O/C	22.3	1.12	58	14.7
FTO/WS_2_/Cs_2_PtI_6_/Cu_2_O/C	23.4	1.13	58.5	15.4

### 3.6 Effect of the back metal contact on the cell performance

**Figure 9:**
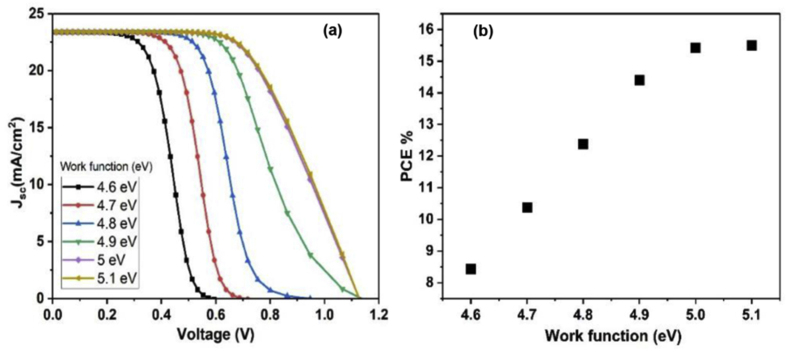
(a) J-V characteristics and (b) PCE for (FTO/WS_2_/Cs_2_PtI_6_/Cu_2_O/metal back contact), with different metal back contacts.

The metal back contact effect on solar cell device performance is studied. The work functions of the selected back contact metals were ranged from 4.6 to 5.1 eV. The J-V characteristics as a function of metal work function values are presented in Figure 9(a). The J-V increased gradually with work function values increase from 4.6 eV to 5 eV. It is observed that the J-V curves are overlapped when the metal back contact work function equal 5 and 5.1 eV, and it can be explained by the consistency of the band structures of the back contact with Cu_2_O HTL material which in turns allows better carriers transfer. In terms of PCE, Figure 9(b) shows the change of PCE with different metal back contacts. It can be observed that the PCE enhanced with metal back contacts with work functions ranged from 4.6 eV to 5 eV. Meanwhile, for the work function of carbon material (5 eV) and noble Au (5.1 eV), there is only a slight change in PCE. The suggested metal back contact is carbon with work function of 5 eV as it is a cheap available material and achieve high PCE. Regarding the above study, it can be concluded that the most optimum structure in terms of materials for Cs_2_PtI_6_–based solar cell is FTO/WS_2_/Cs_2_PtI_6_/Cu_2_O/Carbon.

### 3.7 Optimization of absorber layer thickness

**Figure 10:**
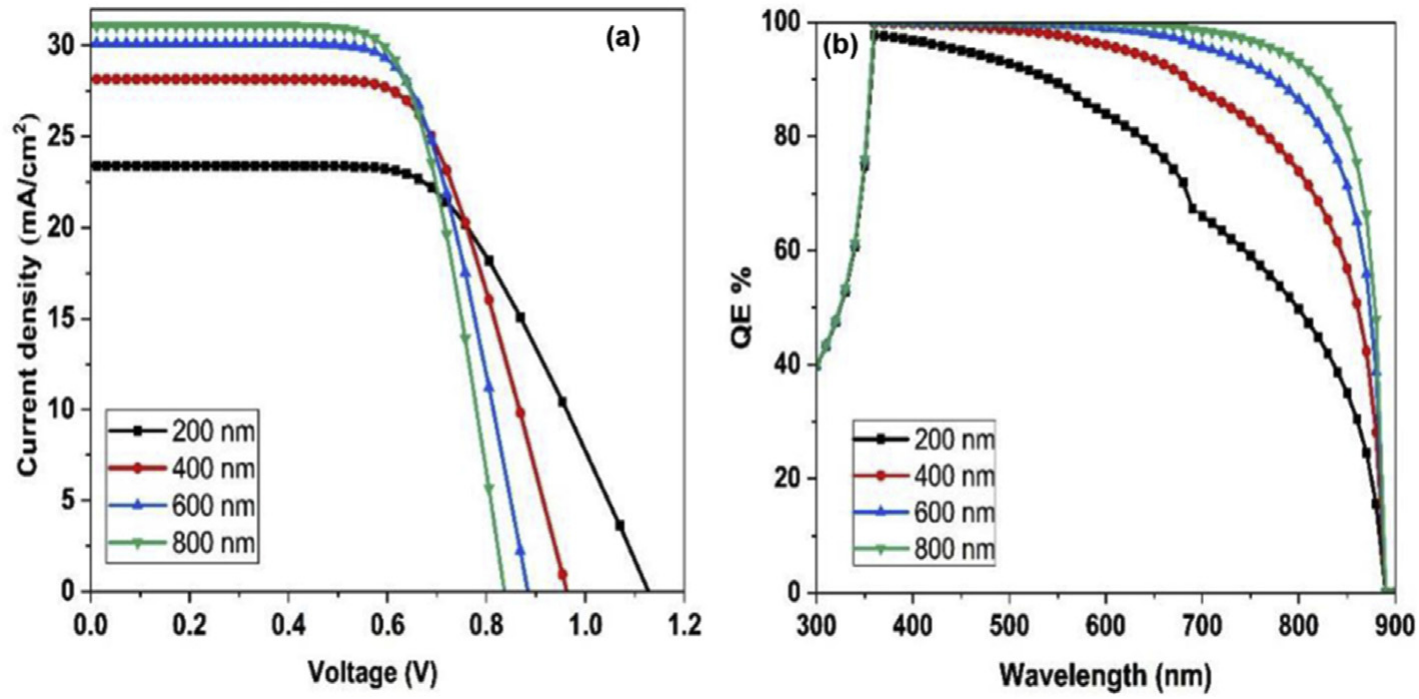
(a) J-V diagram with varying Cs_2_PtI_6_ thickness, (b) QE of Cs_2_PtI_6_ based solar cell with different thickness.

The thickness of active layer has an important influence on the solar cell device performance. Larger thickness allows for more photo absorption of light; however, the diffusion length of carriers may cause recombination of electron-hole pairs and affect the PCE, therefore a thickness optimization of the absorber layer should be done. Figure 10 shows the effect of changing the thickness of the Cs_2_PtI_6_ active layer on the performance of the optimized device structure (FTO/WS_2_/Cs_2_PtI_6_/Cu_2_O/Carbon) perovskite solar cell. The simulated thicknesses ranged from 200 to 800 nm. From the J-V curves, increasing the thickness of the active layer lowers the V_oc_ values and enhances the J_sc_ values as shown in Figure 10(a). Increasing the active layer thickness gradually has a good enhancement on the Jsc of the PSC solar cell. This can be explained by the high photon absorption. As thickness exceeds 400 nm, a high carrier recombination rates will affect the PSC performance. The Jsc will saturate and causes an overall slight change of PCE of solar cell. For QE values, Figure 10(b), the increasing the thickness of Cs_2_PtI_6_ layer enhances the QE of the device which may be due to the high photon absorption accompanied with large thickness. Table 5 summarizes the solar cell parameters with increasing the thickness of Cs_2_PtI_6_ layer from 200 nm to 800 nm. The optimum thickness of Cs_2_PtI_6_ layer is 400 nm in terms of PCE and less material usage.

**Table 5:** Solar cell parameters with different Cs_2_PtI_6_ layer thickness.

Cs_2_PtI_6_ layer thickness	J_sc_ (mA/cm^2^)	V_oc_ (volts)	FF %	PCE %
200 nm	23.4	1.13	58.5	15.4
400 nm	28.1	1.1	52.4	16.3
600 nm	30.1	1.08	49.6	16.2
800 nm	31	1.07	48.3	16

### 3.8 Effects of acceptor carrier and defect density concentrations

**Figure 11:**
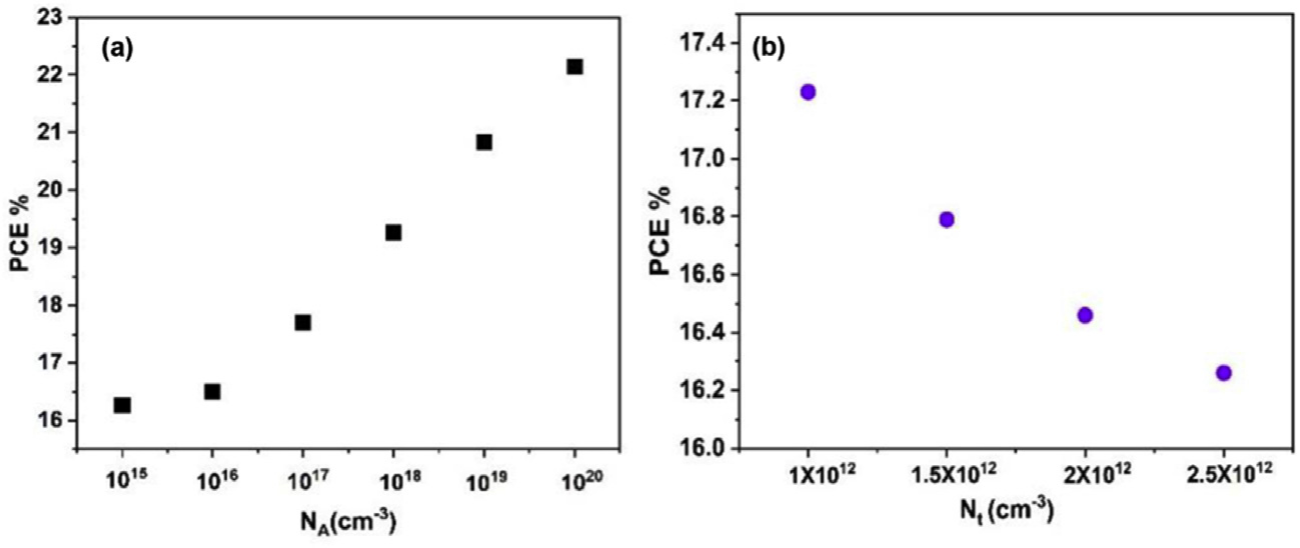
(a) N_A_, (b) N_t_ Vs. the PCE of the optimized solar cell structure FTO/WS_2_/Cs_2_PtI_6_/Cu_2_O/Carbon with different Cs_2_PtI_6_.

Figure 11 shows the effect of doping concentration N_A_ and defect density N_t_ of absorber layer change in PCE. The optimized structure of Cs_2_PtI_6_-based perovskite solar cell (FTO/WS_2_/Cs_2_PtI_6_ (400 nm)/Cu_2_O/Carbon) is simulated with different N_A_, N_t_ values. The PCE increases from 16.3% to up to 22.2 % using N_A_ of 10^20^ cm^−3^ as shown in Figure 11(a). The impact of N_A_ on PCE can be explained Eqs. (10) and (11). The increase of N_A_ decreases the saturation current I_0_ and in turns increase Voc. On the other hand, as Voc increase Jsc decrease.

(10) $$I_{0=}qn_{i}^{2}\left(\frac{D_{n}}{L_{n}N_{A}}+\frac{D_{p}}{L_{p}N_{D}}\right)$$

(11) $$V_{oc}=\frac{KT}{q}\text{ ln}\left(\frac{I_{l}}{I_{0}}+1\right)$$

The defect density results from the fabrication methods of perovskite solar cell based on one or two step spin on solution methods. Fewer defect density concentrations cause higher power conversion efficiencies of perovskite solar cell [82]. The higher values of defect density concentration in the material cause high recombination rates in the material which influences PCE. For these reasons, the effect of defect density concentration on PCE should be studied. According to Eq. (9), the decrease in N_t_ value leads to increase in carrier lifetime and diffusion length which will reduce the recombination rates in Cs_2_PtI_6_ material and increase the PCE of the cell. The impact of defect density of the Cs_2_PtI_6_ layer on the PCE is shown in Figure 11(b). With the appropriate control of the defect density concentration level, the PCE can exceed 17% with defect density of 1 × 10^12^cm^−3^.

The optimized structure of Cs_2_PtI_6_ based perovskite solar cell can be assumed from the simulation study as: FTO/WS_2_/Cs_2_PtI_6_ (400 nm)/Cu_2_O/Carbon and is shown in Figure S3 (a). The doping acceptor concentration of Cs_2_PtI_6_ layer can be estimated to be 10^20^ cm^−3^ and the best defect density concentration levels N_t_ of 1×10^12^ cm^−3^. Figure S3 (b) shows the J-V characteristics diagram of the solar cell structure FTO/WS_2_/Cs_2_PtI_6_ (400 nm)/Cu_2_O/Carbon. The device short circuit current density (Jsc) of 28.15 mA/cm^2^, open circuit voltage (V_oc_) of 1.3 V, power conversion efficiency (PCE) of 22.4 % and fill factor (FF) of 61%.

## 4 Conclusion

The geometrical structures, electronic, and optical properties of the mixed-halide Cs_2_PtI_6__−__x_Br_x_ (x = 0, 2, 4, and 6) were investigated by DFT calculation. The HSE06 functional has successfully predicted the bandgap values of Cs_2_PtI_6_. The bandgap values range from 1.4 to 2.6 eV by increasing the Br^−^ doping content. An investigation has been done on Cs_2_PtI_6_, Cs_2_PtI_4_Br_2_, Cs_2_PtI_2_Br_4,_ and Cs_2_PtBr_6_ in the application of photocatalytic water splitting and CO_2_ reduction. The calculation results showed that the four materials can split water to H_2_ and O_2_. For CO_2_ reduction, the four materials can effectively convert CO_2_ to CH_4_OH and CH_4_. Besides, Cs_2_PtI_6_ and Cs_2_PtI_4_Br_2_ can convert CO_2_ to HCHO. For photovoltaic application, Cs_2_PtI_6_ material is suitable for single-junction solar cells, while other Br^−^ doping materials are suitable for top cell in tandem solar cells with suitable engineering. A device modelling of Cs_2_PtI_6_ based all inorganic solar cell is provided using SCAPS-1D simulator. Different HTL and ETL inorganic materials are simulated. The selection of different ETLs and HTLs is based on the good band alignment matching between them and the band edges LUMO and HOMO of Cs_2_PtI_6_ material. Based on the simulation results, Cu_2_O HTL material has better performance than other HTL materials, therefore it is deemed as the best HTL material for Cs_2_PtI_6_ PSC. Different promising ETLs like CdS, IGZO, ZnSe and WS_2_ were simulated. WS_2_ as an ETL material shows better performance for the Cs_2_PtI_6_ PSC. Further device optimization is done on the device with Cu_2_O HTL and WS_2_ ETL. Different metal work functions are tested for the device simulation. Simulation results showed that the device reveals relatively high PCE with metal work function higher than 4.9 eV. The optimization is expanded to simulate different Cs_2_PtI_6_ absorber thickness. According to simulation, device with 400 nm absorber layer thickness has the better PCE. The doping concentration of 10^20^ cm^−3^ allows high performance of the device. The smaller values of defect density are essential for high performance device. The device has the best performance with defect density concentration of 1×10^12^ cm^−3^. The best possible efficiency of 17.2% for FTO/WS_2_/Cs_2_PtI_6_ (400 nm)/Cu_2_O/Carbon can be achieved. The results can guide for more investigation and optimization of lead free all inorganic perovskite solar cell.

## Declarations

### Author contribution statement

Hadeer H. AbdElAziz: Performed the experiments; Analyzed and interpreted the data; Wrote the paper.

Mohamed Taha, Laila Saad: Conceived and designed the experiments; Analyzed and interpreted the data.

Waleed M.A. El Rouby, M.H. Khedr: Contributed reagents, materials, analysis tools or data; Revised the paper.

### Funding statement

This research did not receive any specific grant from funding agencies in the public, commercial, or not-for-profit sectors.

### Data availability statement

Data included in article/supp. material/referenced in article.

### Declaration of interest’s statement

The authors declare no conflict of interest

### Additional information

No additional information is available for this paper.
