# Supplementary material for: Evaluating the performance of Cs2PtI6−xBrx for photovoltaic and photocatalytic applications using first-principles study and SCAPS-1D simulation
Source: Heliyon. 2022 Sep 28;8(10):e10808. doi: 10.1016/j.heliyon.2022.e10808 (PMC9530494; doi:10.1016/j.heliyon.2022.e10808)
Supplement: supplementary file [file mmc1.docx]

Evaluating the performance of Cs_2_PtI_6-x_Br_x_ for photovoltaic and photocatalytic applications using first-principles study and SCAPS simulation

*Hadeer H. AbdElAziz^1^,* [*Mohamed Taha*](https://pubs.rsc.org/en/results?searchtext=Author%3AMohamed%20Taha)*^1^, Waleed M.A. El Rouby^1^, M.H. Khedr^1^, and Laila Saad^2,*^*

^1^ Material Science and Nanotechnology Department, Faculty of Postgraduate Studies for Advanced Sciences (PSAS), Beni-Suef University, 62511 Beni-suef, Egypt.

^2^ Department of Renewable Energy Science and Engineering, Faculty of Postgraduate Studies for Advanced Sciences (PSAS), Beni-Suef University, 62511 Beni-suef, Egypt.

* Corresponding author: Dr. Laila Saad

E-mail: Laila.hamam@psas.bsu.edu.eg

1. **DFT study**


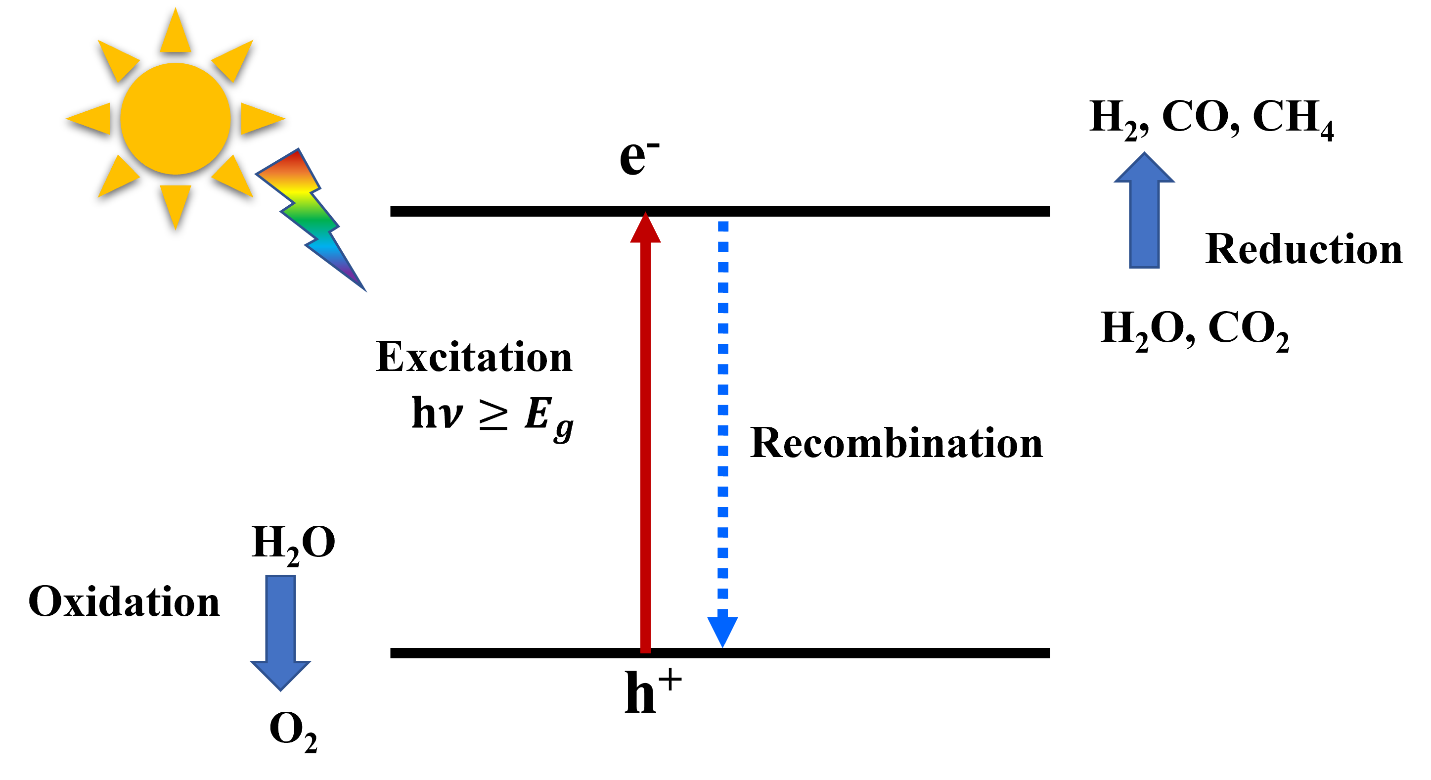


Figure S1 Photocatalytic processes on semiconductor NCs involving the exciton generation, electron-hole pairs separation, and charge carriers’ transfer. The overpotentials, DE, provide the driving force for the charge carriers transfer to the electron and hole acceptor

Table S1 Estimations of electron negativity, electron affinity and band positions of Cs_2_PtI_6-x_Br_x_

|  | **Cs_2_PtI_6_** | **Cs_2_PtI_4_Br_2_** | **Cs_2_PtI_2_Br_4_** | **Cs_2_PtBr_6_** |
| --- | --- | --- | --- | --- |
| Electron negativity$\chi^{M}$ | 5 | 5.1 | 5.3 | 5.4 |
| Electron affinity $\chi$ | 4.3 | 4.44 | 4.6 | 4.7 |
| E_CB_ | -0.12 | -0.09 | -0.02 | -0.03 |
| E_VB_ | 1.3 | 1.5 | 1.7 | 2.3 |

1. **Numerical study**

Table S2 Device parameter for the *SCAPS-1D* simulation *of Cs_2_PtI_6_*

| Parameter | Value |
| --- | --- |
| Cell Temperature T° (K) | 298.15 |
| R_s_ (Ω.cm^2^) | 14 |
| Frequency (Hz) | 1×10^6^ |
| Spectrum | Reference air mass 1.5 (AM1.5) |
| Front contact |  |
| Work Function (eV) | Flat bands |
| Surface Recombination velocity electrons (cm/s) | 10^5^ |
| Surface Recombination velocity holes (cm/s) | 10^7^ |
| Metal contact |  |
| Work Function (eV) | Varied |
| Surface Recombination velocity electrons (cm/s) | 10^7^ |
| Surface Recombination velocity holes (cm/s) | 10^5^ |


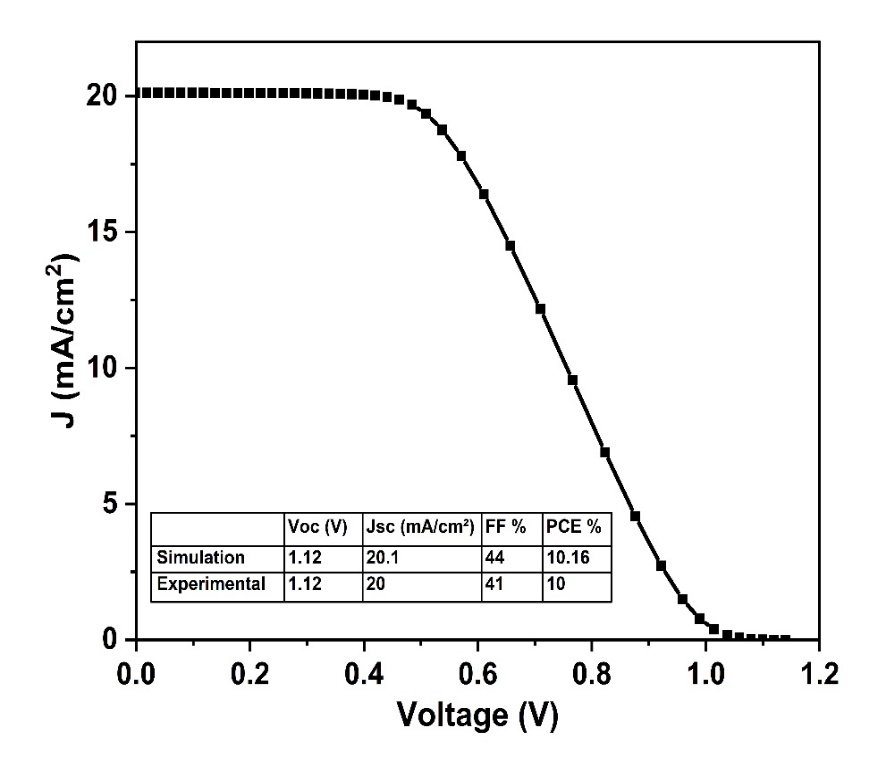

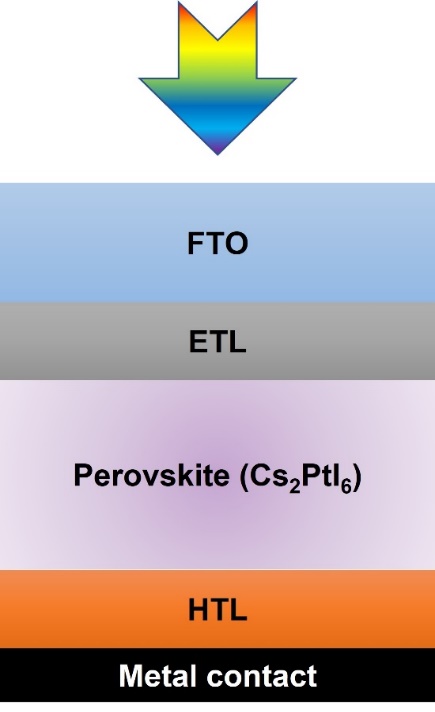


**(a)**

**(b)**

Figure S2 (a) N-I-P planar structure FTO/ETL/Cs_2_PtI_6_/HTL/Metal solar cell, (b) J-V diagram calculated from reference cell example in supporting information Table S1-S3

Table S3 Material properties of FTO, Cs_2_PtI_6_ and IDLs

|  | **FTO (500 nm)**  [1] | **Front IDL (5 nm)** | **Cs_2_PtI_6_ (200- 800 nm)**  [2] | **Back IDL (5 nm)** |
| --- | --- | --- | --- | --- |
| Electron affinity (eV), χ | 4 | 4 | 4 | 4 |
| Relative permittivity, ɛ | 9 | 16.72 | 16.72 | 16.72 |
| E_g_ (eV) band gap | 3.5 | 1.4 | 1.4 | 1.4 |
| Electron mobility (cm^2^v^-1^s^-1^), µ_e_ | 20 | 310 | 310 | 310 |
| Hole mobility (cm^2^v^-1^s^-1^), µ_h_ | 10 | 62 | 62 | 62 |
| Donor density (cm^-3^), N_D_ | 1×10^18^ | 0 | 0 | 0 |
| Acceptor density (cm^-3^), N_A_ | 0 | 10^15^ | 10^15^ | 10^15^ |
| Effective conduction band density (cm^-3^), N_c_ | 2.2×10^18^ | 2.2×10^18^ | 2.2×10^18^ | 2.2×10^18^ |
| Effective valence band density (cm^-3^), N_v_ | 1.8×10^19^ | 1.8×10^19^ | 1.8×10^19^ | 1.8×10^19^ |
| Defect density (cm^-3^), N_t_ | 1×10^15^ | 2.5×10^12^ | 2.5X10^12^ | 2.5×10^12^ |

Table S4 Material properties of different HTLs

|  | **Cu_2_O** [3] | **CuI** [4] | **MoO_3_** [5] | **Spiro-OMeTAD** [6] |
| --- | --- | --- | --- | --- |
| Electron affinity (eV), χ | 3.2 | 2.1 | 2.3 | 2.4 |
| Relative permittivity, ɛ | 7.11 | 6.5 | 12.5 | 3 |
| E_g_ (eV) band gap | 2.17 | 3.1 | 3 | 3 |
| Electron mobility (cm^2^v^-1^s^-1^), µ_e_ | 200 | 100 | 25 | 2 ×10^-4^ |
| Hole mobility (cm^2^v^-1^s^-1^), µ_h_ | 80 | 43.9 | 100 | 2 ×10^-4^ |
| Acceptor density (cm^-3^), N_A_ | 1×10^18^ | 1×10^18^ | 1×10^18^ | 1×10^18^ |
| Effective conduction band density (cm^-3^), N_c_ | 2.2×10^18^ | 2.2×10^18^ | 2.2×10^18^ | 2.2×10^18^ |
| Effective valence band density (cm^-3^), N_v_ | 1.8×10^19^ | 1.8×10^19^ | 1.8×10^19^ | 1.8×10^19^ |
| Defect density (cm^-3^), N_t_ | 1×10^15^ | 1×10^15^ | 1×10^15^ | 1×10^15^ |

Table S5 Material properties of different ETLs

|  | **CdS** [7] | **IGZO** [8]**,**[9] | **ZnSe** [10] | **WS_2_** [11] |
| --- | --- | --- | --- | --- |
| Electron affinity (eV), χ | 4.2 | 4.16 | 4.09 | 3.95 |
| Relative permittivity, ɛ | 10 | 10 | 8.6 | 13.6 |
| E_g_ (eV) band gap | 2.4 | 3.050 | 2.81 | 1.8 |
| Electron mobility (cm^2^v^-1^s^-1^), µ_e_ | 100 | 15 | 400 | 100 |
| Hole mobility (cm^2^v^-1^s^-1^), µ_h_ | 25 | 0.1 | 100 | 100 |
| Donor density (cm^-3^), N_D_ | 1×10^18^ | 1×10^18^ | 1×10^18^ | 1×10^18^ |
| Effective conduction band density (cm^-3^), N_c_ | 2.2×10^18^ | 5×10^18^ | 2.2×10^18^ | 1×10^18^ |
| Effective valence band density (cm^-3^), N_v_ | 1.8×10^19^ | 5×10^18^ | 1.8×10^19^ | 1×10^18^ |
| Defect density (cm^-3^), N_t_ | 1×10^19^ | 1×10^15^ | 1×10^15^ | 1×10^15^ |

Table S6 The metal back contact work function

| **Metal** | **Work function (eV)** |
| --- | --- |
| Copper (Cu) | 4.6 [12] |
| Silver (Ag) | 4.7~4.8 [13], [14] |
| Graphene Oxide (GO) | 4.9 [15] |
| Carbon (C) | 5 [16] |
| Gold (Au) | 5.1[17] |


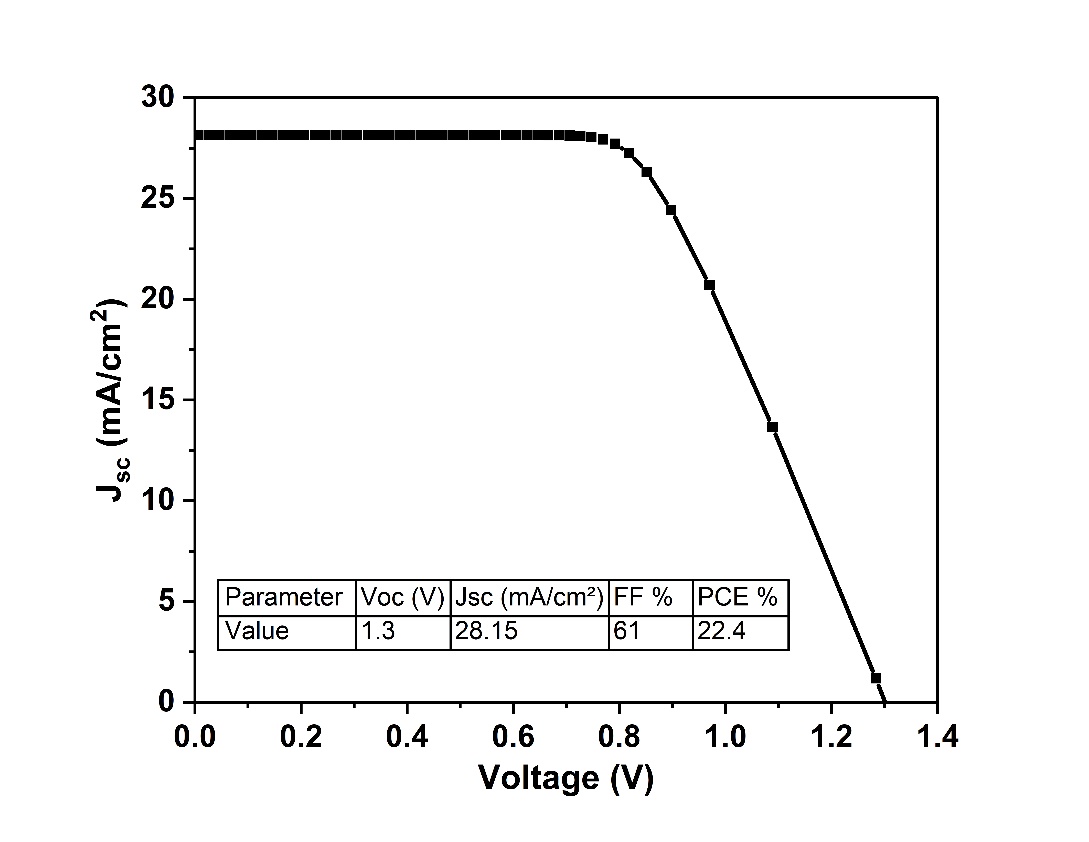

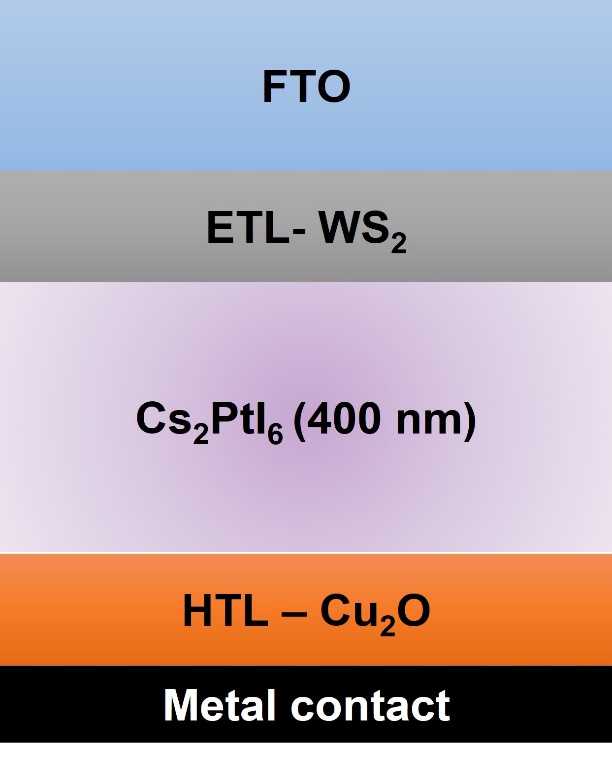


**(b)**

**(a)**

Figure S3 (a) Optimized structure, (b) J-V characteristics diagram of optimized solar cell structure

# References

[1] S. Abdullahi *et al.*, “Optical Characterization of Fluorine doped Tin Oxide (FTO) thin films deposited by spray pyrolysis technique and annealed under Nitrogen atmosphere,” *Int. J. Innov. Appl. Stud.*, vol. 9, no. 2, pp. 947–955, 2014.

[2] D. Schwartz *et al.*, “Air Stable, High-Efficiency, Pt-Based Halide Perovskite Solar Cells with Long Carrier Lifetimes,” *Phys. Status Solidi - Rapid Res. Lett.*, vol. 14, no. 8, pp. 1–8, 2020, doi: 10.1002/pssr.202000182.

[3] M. I. Hossain, F. H. Alharbi, and N. Tabet, “Copper oxide as inorganic hole transport material for lead halide perovskite based solar cells,” *Sol. Energy*, vol. 120, pp. 370–380, 2015, doi: 10.1016/j.solener.2015.07.040.

[4] A. A. Kanoun, M. B. Kanoun, A. E. Merad, and S. Goumri-Said, “Toward development of high-performance perovskite solar cells based on CH3NH3GeI3 using computational approach,” *Sol. Energy*, vol. 182, no. June 2018, pp. 237–244, 2019, doi: 10.1016/j.solener.2019.02.041.

[5] W. W. Li, W. W. Li, Y. Feng, and C. Yang, “Numerical analysis of the back interface for high efficiency wide band gap chalcopyrite solar cells,” *Sol. Energy*, vol. 180, no. December 2018, pp. 207–215, 2019, doi: 10.1016/j.solener.2019.01.018.

[6] X. Gu, Y. Li, Y. Mu, M. Zhang, T. Lu, and P. Wang, “FeCl3 as a low-cost and efficient p-type dopant of Spiro-OMeTAD for high performance perovskite solar cells,” *RSC Adv.*, vol. 8, no. 17, pp. 9409–9413, 2018, doi: 10.1039/c8ra00243f.

[7] L. I. Nykyruy, R. S. Yavorskyi, Z. R. Zapukhlyak, G. Wisz, and P. Potera, “Evaluation of CdS/CdTe thin film solar cells: SCAPS thickness simulation and analysis of optical properties,” *Opt. Mater. (Amst).*, vol. 92, no. December 2018, pp. 319–329, 2019, doi: 10.1016/j.optmat.2019.04.029.

[8] N. Lakhdar and A. Hima, “Electron transport material effect on performance of perovskite solar cells based on CH3NH3GeI3,” *Opt. Mater. (Amst).*, vol. 99, no. October 2019, p. 109517, 2020, doi: 10.1016/j.optmat.2019.109517.

[9] F. Azri, M. Labed, A. F. Meftah, N. Sengouga, and A. M. Meftah, “Optical characterization of a-IGZO thin film for simulation of a-IGZO(n)/µ-Si(p) heterojunction solar cell,” *Opt. Quantum Electron.*, vol. 48, no. 8, pp. 1–16, 2016, doi: 10.1007/s11082-016-0660-5.

[10] J. Adeyemi Owolabi, M. Yusuf Onimisi, J. Amuchi Ukwenya, A. Bulus Bature, and U. Raphael Ushiekpan, “Investigating the Effect of ZnSe (ETM) and Cu2O (HTM) on Absorber Layer on the Performance of Pervoskite Solar Cell Using SCAPS-1D,” *Am. J. Phys. Appl.*, vol. 8, no. 1, p. 8, 2020, doi: 10.11648/j.ajpa.20200801.12.

[11] K. Sobayel *et al.*, “A comprehensive defect study of tungsten disulfide (WS2) as electron transport layer in perovskite solar cells by numerical simulation,” *Results Phys.*, vol. 12, no. December 2018, pp. 1097–1103, 2019, doi: 10.1016/j.rinp.2018.12.049.

[12] E. W. J. Mitchell, J. W. Mitchell, and N. F. Mott, “The work functions of copper, silver and aluminium,” *Proc. R. Soc. London. Ser. A. Math. Phys. Sci.*, vol. 210, no. 1100, pp. 70–84, Dec. 1951, doi: 10.1098/rspa.1951.0231.

[13] A. W. Dweydari and C. H. B. Mee, “Work function measurements on (100) and (110) surfaces of silver,” *Phys. status solidi*, vol. 27, no. 1, pp. 223–230, Jan. 1975, doi: https://doi.org/10.1002/pssa.2210270126.

[14] P. A. Anderson, “A New Technique for Preparing Monocrystalline Metal Surfaces for Work Function Study. The Work Function of Ag(100),” *Phys. Rev.*, vol. 59, no. 12, pp. 1034–1041, Jun. 1941, doi: 10.1103/PhysRev.59.1034.

[15] E. Stratakis, K. Savva, D. Konios, C. Petridis, and E. Kymakis, “Improving the efficiency of organic photovoltaics by tuning the work function of graphene oxide hole transporting layers,” *Nanoscale*, vol. 6, no. 12, pp. 6925–6931, 2014, doi: 10.1039/c4nr01539h.

[16] M. Shiraishi and M. Ata, “Work function of carbon nanotubes,” *Carbon N. Y.*, vol. 39, no. 12, pp. 1913–1917, 2001, doi: https://doi.org/10.1016/S0008-6223(00)00322-5.

[17] C. Li *et al.*, “Effect of the work function of gate electrode on hysteresis characteristics of organic thin-film transistors with Ta2O5/polymer as gate insulator,” *Org. Electron.*, vol. 10, no. 5, pp. 948–953, 2009, doi: https://doi.org/10.1016/j.orgel.2009.05.001.
